# Large rectal Tubulovillous adenoma leading to McKittrick–Wheelock syndrome

**DOI:** 10.1093/omcr/omaf267

**Published:** 2025-12-26

**Authors:** Mohamad Bahrou, Muhammed Yaman Swied, Ayesha Ali, Elizabeth Tatum

**Affiliations:** Department of Medicine, Southern Illinois University School of Medicine, 801 N. Rutledge St., Springfield, IL 62702, United States; Department of Medicine, Southern Illinois University School of Medicine, 801 N. Rutledge St., Springfield, IL 62702, United States; Department of Medicine, Southern Illinois University School of Medicine, 801 N. Rutledge St., Springfield, IL 62702, United States; Department of Medicine, Southern Illinois University School of Medicine, 801 N. Rutledge St., Springfield, IL 62702, United States

**Keywords:** AKI, diarrhea, electrolyte imbalance, McKittrick–Wheelock syndrome, tubulovillous adenoma

## Abstract

McKittrick–Wheelock syndrome (MWS) is a rare condition characterized by chronic diarrhea caused by colorectal villous adenoma, which can lead to severe hyponatremia, hypokalemia, and kidney injury. We present a case of MWS resulting from a 9.6 cm rectal tubulovillous adenoma, which caused significant electrolyte abnormalities and kidney injury. While MWS has been documented in association with villous adenomas, there is limited literature on its occurrence with tubulovillous adenomas, as seen in our case.

## Introduction

McKittrick–Wheelock syndrome (MWS) is a rare condition first described by McKittrick and Wheelock in 1954. This condition is characterized by secretory diarrhea caused by villous adenoma in the rectum or rectosigmoid colon, leading to pre-renal kidney injury and electrolyte imbalance. A total of 257 confirmed cases have been reported, according to the largest and most recent systematic review conducted in 2018 [1]. We report a case of a 45-year-old male who presented with chronic diarrhea, severe electrolyte abnormalities, and AKI secondary to a large secretory rectal tubulovillous adenoma consistent with MWS. We aim to highlight the clinical importance of early recognition and treatment of MWS to prevent life-threatening electrolyte imbalances and renal failure.

## Case report

**Figure 1:**
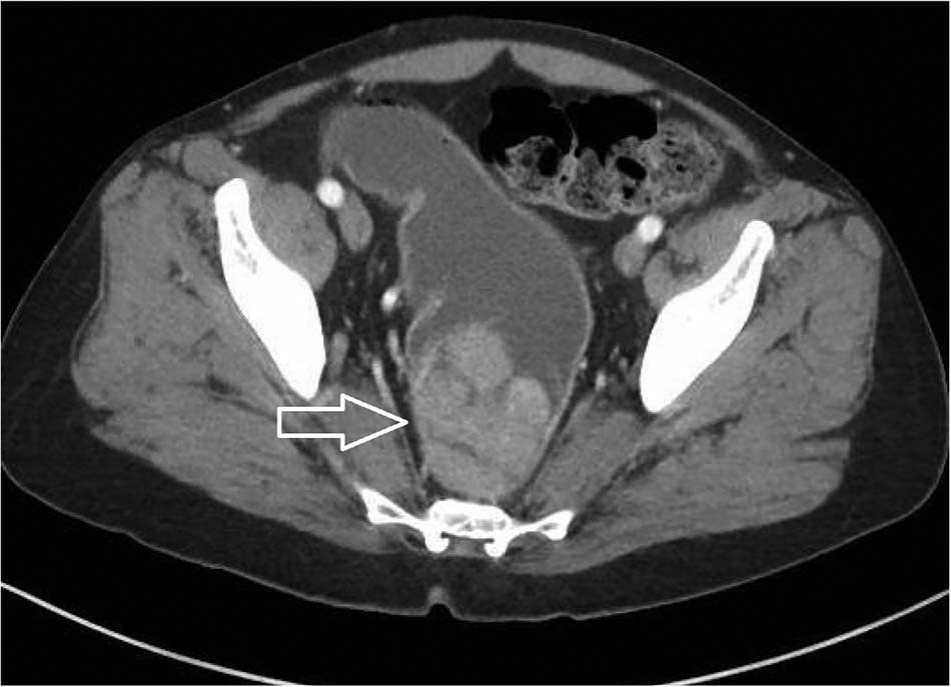
Axial section of the abdominal and pelvic CT showing large villous rectal mass measuring 9.6 cm (white arrow).

**Figure 2:**
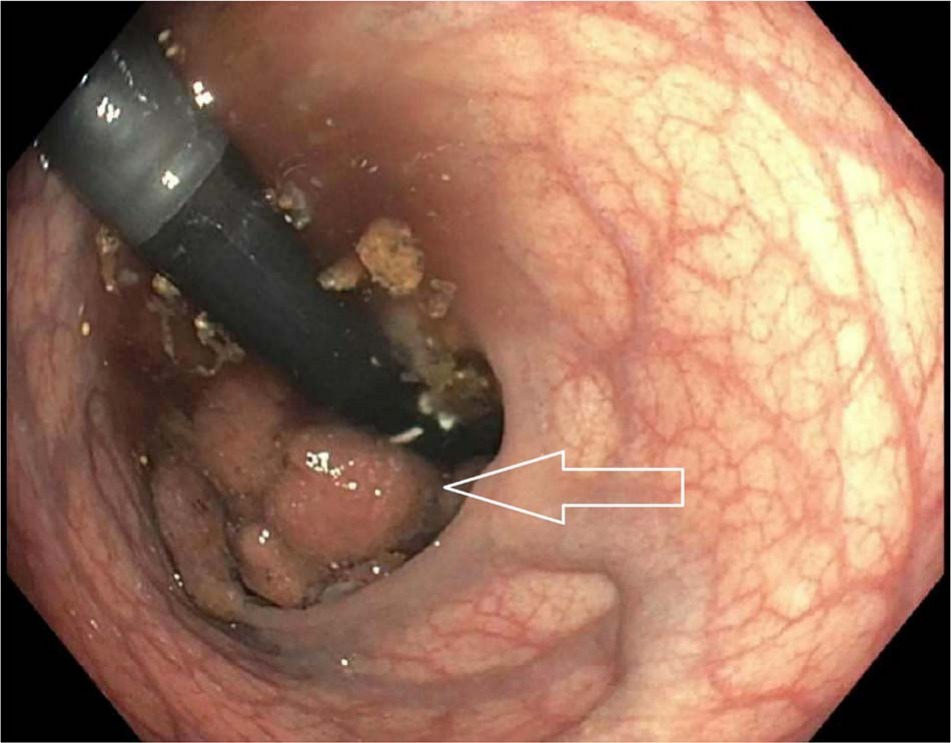
Colonoscopy showing distal rectal polypoid mass (white arrow).

**Figure 3:**
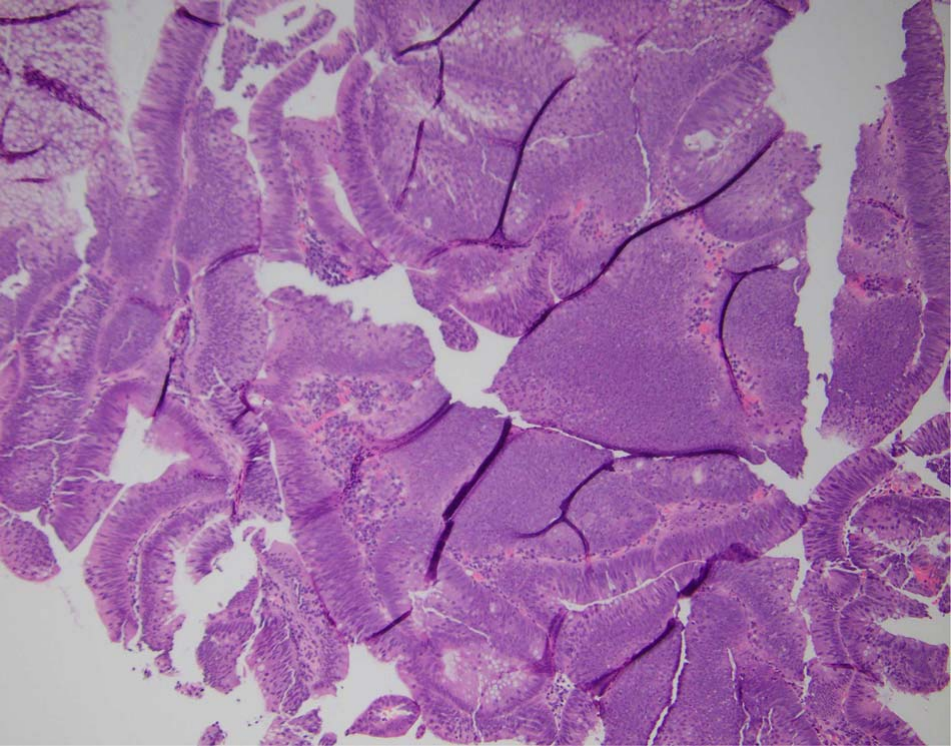
Hematoxylin and eosin stain demonstrating a tubulovillous adenoma without dysplasia (×40 magnification).

A 45-year-old male presented to the emergency department for evaluation of generalized weakness, fatigue, and diarrhea that had persisted for one year. Laboratory results revealed severe AKI and hyponatremia, with a serum creatinine level of 9.9 mg/dL, serum sodium of 103 mmol/L, serum chloride of 55 mmol/L, and serum potassium of 2.8 mmol/L, alongside a high anion gap of 37. Urine studies showed an osmolality of 286 mOsm/kg and urine sodium of less than 20 mEq/L, indicating a hypovolemic etiology likely due to diarrhea. The patient received aggressive intravenous fluid resuscitation and electrolyte replacement. After the initial resolution of AKI and improvement in hyponatremia, the patient experienced a rapid deterioration in renal function, acid–base status, and electrolyte levels after discontinuing intravenous fluids. Repeat urine studies remained consistent with a hypovolemic etiology. Due to persistent diarrhea despite loperamide use, along with leukocytosis and low-grade fevers, abdominal and pelvic computed tomography was performed, which revealed a 9.6 cm villous rectal mass with significant vascularity and arterial and portal venous collaterals surrounding and within the mass (Fig. 1). The CT also showed evidence of mild diverticulitis, which was treated with antibiotics. Based on the laboratory data and imaging findings, the patient was suspected to have MWS. Laboratory abnormalities improved with aggressive IV hydration and encouragement of oral hydration with electrolyte-containing beverages. Colonoscopy was subsequently performed, revealing a distal rectal polypoid mass measuring 9.6 cm (Fig. 2). Biopsies of the rectal mass demonstrated a tubulovillous adenoma with no evidence of dysplasia (Fig. 3). Given the large size of the rectal adenoma, the patient was scheduled for rectal resection with a diverting loop ileostomy. However, the patient was unable to undergo surgery due to lack of insurance coverage and was subsequently lost to outpatient follow-up.

## Discussion

MWS is a rare clinical condition characterized by secretory diarrhea due to a colorectal tumor, most commonly a large villous adenoma. These tumors contain mucin-secreting epithelial cells that actively transport sodium and potassium into the intestinal lumen, creating an osmotic gradient that draws large volumes of water into the bowel. This mechanism results in profuse, watery diarrhea, with symptom severity typically correlating with tumor size. The resulting volume depletion can cause profound electrolyte disturbances, including hyponatremia (<125 mEq/L), hypokalemia (<3.5 mEq/L), and hypochloremia (<98 mEq/L), often accompanied by metabolic acidosis and pre-renal azotemia. Without timely recognition and intervention, these abnormalities can progress to AKI and life-threatening complications.

A systematic review of 257 cases of MWS reported a median symptom duration of 24 months before hospital presentation [1]. The majority of tumors were located distal to the splenic flexure, with 60.5% involving the rectum, and a median tumor size of 12 cm [1]. Chronic diarrhea was the predominant presenting symptom, followed by nausea and vomiting, highlighting the insidious and progressive nature of the syndrome [1]. Imaging modalities, particularly contrast-enhanced CT scans, are crucial in identifying large colorectal villous adenomas, most commonly located in the rectum or sigmoid colon. Colonoscopy with biopsy is essential to confirm the diagnosis by demonstrating villous or tubulovillous adenomas, which are the source of secretory diarrhea [2].

Management of MWS involves both symptomatic stabilization and definitive tumor resection. Indomethacin and octreotide have been used to reduce fluid loss and correct electrolyte disturbances while awaiting definitive surgery or as an alternative to surgical intervention [3, 4]. Some case reports indicate using indomethacin or octreotide for patients who cannot undergo surgery. However, other studies suggest that these treatments offer no benefits beyond proper fluid and electrolyte replacement. Definitive treatment requires removal of the adenoma, most commonly via surgical resection [1]. Standard approaches include anterior resection with diverting loop ileostomy, followed by ileostomy reversal, a strategy employed in approximately 64.8% of cases. For select patients with smaller or more accessible lesions, less invasive options such as endoscopic resection or brachytherapy may be attempted, despite a higher risk of recurrence. Without treatment, MWS carries a mortality rate approaching 100% due to profound electrolyte derangement and renal failure [5]. However, with timely and appropriate surgical intervention, mortality has been reported as low as 5.2% [1]. In our case, the patient was scheduled for surgical resection but was unable to undergo the procedure due to lack of insurance and was subsequently lost to follow-up. By the time the diagnosis was confirmed on colonoscopy and biopsy, his AKI and electrolyte abnormalities had normalized with intravenous fluid and electrolyte replacement. Additional medical therapy, including indomethacin or octreotide, was not required prior to the planned surgery.

In conclusion, we present a case of a large rectal tubulovillous adenoma leading to MWS. This case highlights the importance of considering MWS in patients with chronic diarrhea, severe electrolyte disturbances, and kidney injury, particularly when a rectal mass is detected on imaging or colonoscopy. Timely recognition is essential to guide appropriate management, which involves aggressive fluid and electrolyte replacement followed by definitive surgical resection.

## References

[1] Orchard MR, Hooper J, Wright JA, McCarthy K. A systematic review of McKittrick-Wheelock syndrome. Ann R Coll Surg Engl 2018;100:591–597. Published online: October 16, 2018. 10.1308/rcsann.2018.0184 PMC6204505 30322287

[2] Vertolli U, De Giorgi L, Melega E. et al. Severe diarrhea and the McKittrick and Wheelock syndrome. QJM. 2023;116:310–2. 10.1093/qjmed/hcac255 36355562

[3] Fernández-López F, Paredes-Cotore JP. McKittrick-Wheelock syndrome - prolapsed giant villous adenoma of the rectum. Rev Esp Enferm Dig 2013;105:309–10. 10.4321/s1130-01082013000500017 23971670

[4] Nakhla SG, Murakami TT, Sundararajan S. Poorly differentiated neuroendocrine tumor of the rectum coexistent with Giant rectal villous adenoma presenting as McKittrick-Wheelock syndrome. Case Rep Oncol Med 2015;2015:1–3. 10.1155/2015/242760 PMC4670721 26682079

[5] Emrich J, Niemeyer C. Das sezernierende villöse Adenom als seltene Ursache einer akuten Niereninsuffizienz [the secreting villous adenoma as a rare cause of acute renal failure]. Med Klin (Munich) 2002;97:619–23. 10.1007/s00063-002-1203-3 12386796

